# Immunolocalization of Matrix Metalloproteinases 2 and 9 and Their Inhibitors in the Hearts of Rats Treated with Immunosuppressive Drugs—An Artificial Intelligence-Based Digital Analysis

**DOI:** 10.3390/biomedicines12040769

**Published:** 2024-03-30

**Authors:** Aleksandra Wilk, Małgorzata Król, Kajetan Kiełbowski, Estera Bakinowska, Kamila Szumilas, Anna Surówka, Karolina Kędzierska-Kapuza

**Affiliations:** 1 Department of Histology and Embryology, Pomeranian Medical University, 70-111 Szczecin, Poland; aleksandra.wilk@pum.edu.pl (A.W.); malgorzatakrol246@gmail.com (M.K.); esterabakinowska@gmail.com (E.B.); 2 Department of Physiology, Pomeranian Medical University, 70-111 Szczecin, Poland; kamila.szumilas@pum.edu.pl; 3 Department of Plastic, Endocrine and General Surgery, Pomeranian Medical University, 72-010 Szczecin, Poland; anna.surowka@pum.edu.pl; 4 Department of Gastroenterological Surgery and Transplantology, Center of Postgraduate Medical Education in Warsaw, 137 Wołoska St., 02-507 Warsaw, Poland; karolina.kedzierska@gmail.com

**Keywords:** immunosuppression, cyclosporine, tacrolimus, rapamycin, mycophenolate, matrix metalloproteinase, tissue inhibitor of matrix metalloproteinase, artificial intelligence

## Abstract

Background: Immunosuppressive agents represent a broad group of drugs, such as calcineurin inhibitors, mTOR inhibitors, and glucocorticosteroids, among others. These drugs are widely used in a number of conditions, but lifelong therapy is crucial in the case of organ recipients to prevent rejection. To further increase the safety and efficacy of these agents, their off-target mechanisms of action, as well as processes underlying the pathogenesis of adverse effects, need to be thoroughly investigated. The aim of this study was to examine the impact of various combinations of cyclosporine/tacrolimus/mycophenolate with rapamycin and steroids (CRG, TRG, MRG), on the morphology and morphometry of rats’ cardiomyocytes, together with the presence of cardiac collagen and the immunoexpression of MMPs and TIMPs. Methods: Twenty-four rats were divided into four groups receiving different immunosuppressive regiments. After six months of treatment, the hearts were collected and analyzed. Results: Cardiomyocytes from the CRG cohorts demonstrated the most pronounced morphological alterations. In addition, chronic immunosuppression reduced the width and length of cardiac cells. However, immunosuppressive therapy did not alter the presence of cardiac collagen fibers. Nevertheless, we observed significant alterations regarding MMP/TIMP homeostasis. Conclusions: Chronic immunosuppression seems to disturb the MMP/TIMP balance in aspects of immunolocalization in the hearts of rats. Further studies are required to analyze other mechanisms and pathways affected by the use of immunosuppressants.

## 1. Introduction

Immunosuppressive therapy is a lifelong strategy to prevent rejection in organ transplant recipients. Multiple drugs with immunosuppressive capabilities are used in clinical practice, such as calcineurin inhibitors (cyclosporine (CsA), tacrolimus), mTOR inhibitors (sirolimus, everolimus), mycophenolate mofetil, or glucocorticosteroids, among others. The role of these agents is to suppress the pathways of the immune system that could trigger an inflammatory response towards graft. These agents can be administered in various combinations and strategies, and different drugs may be used in an induction of therapy and subsequent maintenance immunosuppression [1]. The use of immunosuppressants is associated with certain adverse events, such as hypertension, diabetes mellitus, renal failure, or malignancy, among others [2]. Furthermore, chronic exposure to immunosuppressive agents can promote organ fibrosis, thus negatively affecting its functionality and potentially leading to organ failure or graft rejection [3,4]. Nevertheless, the precise role of the immunosuppressants on the functionality and morphology of organs is not entirely known.

One of the organs that is exposed to the harmful action of immunosuppressive drugs is the heart. The heart is a complex organ that contains multiple cell types, and of note, each of the cells plays an important role under both physiological and pathophysiological conditions [5]. The heart’s wall is composed of endocardium, myocardium, and epicardium. Additionally, the organ is located in a connective tissue bag known as pericardium, which protects the heart against mechanical disturbances. The endocardium is lined with endothelium, while the subendocardiac part, composed of connective tissue, is located below. The second part of the endocardium is a muscular–elastic layer with numerous elastic fibers that are visible in orcein as a dark line. The final and third part is a subendocardium, which contains Purkinje fibers. Purkinje fibers are modified cardiomyocytes, and they are involved in controlling the heart beating. The myocardium is mainly composed of cardiomyocytes, which play a major role in maintaining blood circulation via their spontaneous activity and pumping function. In the aspect of total cardiac cells, cardiomyocytes constitute approximately 30% to 40% [6,7]. Numerous cells exist in the interstitial spaces of the heart wall, and these are known as “non-myocytes”. The non-myocyte group comprises heterogeneous cell lineages, such as those mentioned above, endothelial cells, vascular smooth muscle cells, pericytes, fibroblasts, macrophages, and other types of cells, including cardiac stem or progenitor cells. These cells communicate with each other not only by direct physical contact but also by paracrine signaling [5]. However, it should be emphasized that cardiomyocytes are the cells that are exposed to the action of immunosuppressive drugs. Epicardium constitutes the outer layer of the wall of the heart, and it is composed of connective tissue and adipose tissue, and it is lined with mesothelium.

One of the side effects of long-term usage of immunosuppressive drugs is their effect on MMP/TIMP balance in tissues and organs. Under physiological conditions, endothelial and cardiomyocytes control the secretion of MMPs and their inhibitors (TIMPs—metalloproteinase inhibitors) at a constant level. This allows a balance to be maintained between the degradation and synthesis of components of the circulatory system, including the heart. A disturbed MMP/TIMP balance may lead to improper extracellular matrix remodeling, and this, consequently, may cause the development of pathological alterations associated with heart failure.

The aim of this study was to investigate the impact of various combinations of cyclosporine, tacrolimus, rapamycin, mycophenolate, and steroids on the expression of collagen and enzymes associated with extracellular matrix homeostasis. To increase the reliability and accuracy, we used artificial intelligence (AI)-based digital analysis software to analyze the presence of collagen fibers, as well as to detect protein expression in immunohistochemical (IHC) staining.

## 2. Materials and Methods

### 2.1. Animals

This study was based on the archival material prepared by the Department of Nephrology, Transplantology and Internal Medicine at the Pomeranian Medical University in Szczecin. Male Wistar rats were used in this experiment. The study was approved by the local Ethics Committee for Experiments on Animals (decision number 24/08, 24 November 2008). Briefly, rats in the control group did not receive any drugs, while animals in the study groups received different protocols of oral immunosuppressants. Agents used in this study included tacrolimus (4 mg/kg/day), cyclosporine A (5 mg/kg/day), rapamycin (0.5 mg/kg/day), and glucocorticosteroids (prednisone 4 mg/kg/day), according to the literature recommendation [8,9,10,11], and to the individual weights of rats. Based on the applied drug strategies, the following animal groups were formed: C: control; CRG: cyclosporine A, rapamycin, glucocorticosteroids; TRG: tacrolimus, rapamycin, glucocorticosteroids; and MRG: mycophenolate mofetil, rapamycin, and glucocorticosteroids. The analysis was performed in the myocardium of six rats per group. Animals from the experimental groups received drugs for six months. After three months of study, the rats were weighed, and the dosages were adjusted.

### 2.2. Morphology

The morphology of cardiac tissue was analyzed similarly to the previously published studies [12,13,14]. The samples were fixed in a freshly prepared 4% paraformaldehyde and embedded in paraffin using routine procedures. For the morphological analysis, serial slices (3–4 µm thickness) of samples of the myocardium of control and experimental rats were mounted on poly-L-lysine coated glass slides and stained with hematoxylin and eosin (H-E). To visualize collagen fibers, the slides were stained with Mallory trichrome (Bio-Optica Milano, Milano, Italy). All histochemical reactions were carried out according to the manufacturer’s protocols.

### 2.3. Morphometry

**Figure 1:**
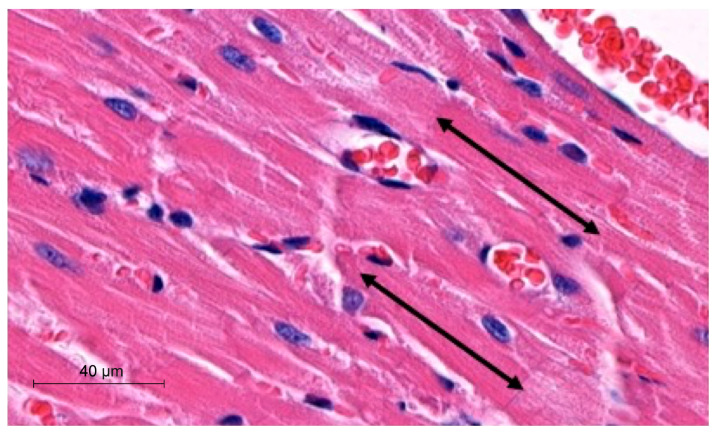
Representative picture of morphometric method with use of SlideViewer; black arrows indicate length of cardiomyocytes.

To perform morphometric analyses, we measured the width and length of cardiomyocytes in µm. The latter parameter was obtained by measuring the distance between two intercalated discs. The analyses were performed with the hematoxylin-eosin (H-E) heart sections using a Slide Viewer application (SlideViewer, 3D Histech, Hungary). Measurements were performed under 40× objective magnification. The length of cardiomyocytes was measured, as demonstrated in Figure 1.

### 2.4. Vascular Density

In order to examine the vascular density of tissue samples, we used the Pattern Quant software version 2.3 (3DHISTECH Kft., Budapest, Hungary). We trained the program to identify the structures of blood vessels on the cross sections of myocardium samples. Specifically, we selected tissue fragments and assigned them to different clusters (blood vessels and tissues other than blood vessels). Subsequently, we randomly selected ten cross-sectioned tissue fragments under 40× objective magnification and scanned them with Pattern Quant. Two independent experts verified the results generated by the software.

### 2.5. Collagen Fibers

**Figure 2:**
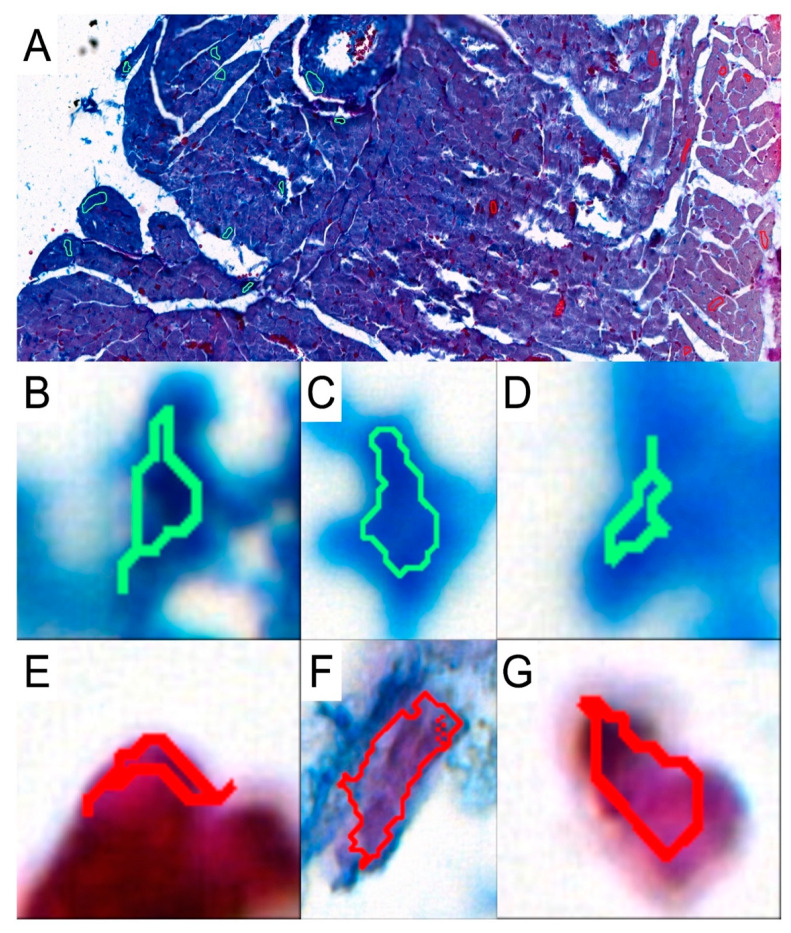
Representative picture of analyzed structures with the use of Pattern Quant software. (**A**): During training, we selected a few examples of collagen-positive areas (green circles) and collagen-negative areas (red circles). Pattern Quant creates a map on the histological specimen with counts classified as follows: (**B**–**D**): examples of collagen-positive areas; (**E**–**G**): examples of collagen-negative areas.

To investigate the presence and abundance of collagen fibers in the obtained specimens, slides were stained with the Mallory trichrome method. To analyze the results, the slides were scanned with a 3DHISTECH Pannoramic MIDI II scanner (Sysmex Polska Sp. z o.o., Warsaw, Poland). We used the Pattern Quant software (3DHISTECH Kft., Budapest, Hungary) to analyze the slides and obtain the percentage of collagen fibers present in the selected areas. We selected appropriate areas and trained the program to identify collagen fibers based on the structure pattern and the color intensity by selecting cluster 1: collagen-positive area and cluster 2: collagen-negative area (Figure 2). Six slides from each group were analyzed. Two independent experts verified and analyzed the results generated by the Pattern Quant software.

### 2.6. Immunohistochemical Analysis

**Figure 3:**
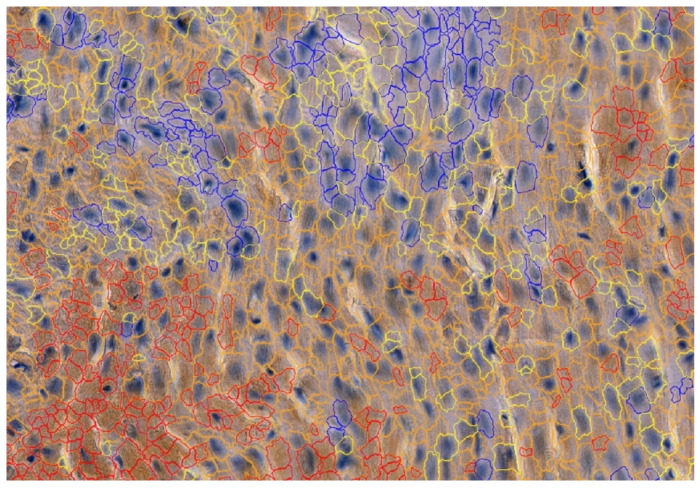
Representative picture of the randomly chosen area with four visible strengths of immunoexpression of analyzed enzymes. Cell Quant creates a map and marks each observation depending on the strength of the immunoexpression of the protein: blue circles: negative expression (−); yellow circles: weak expression (+); orange circles: strong expression (++); red circles: very strong expression (+++).

To detect the expression of MMP-2, MMP-9, TIMP-1 and TIMP-2, the following mouse antibodies were used: anti-MMP-2 at 1:250 (sc-53630; Santa Cruz Biotechnology, Inc., Santa Cruz, CA, USA), mouse anti-MMP-9 at 1:250 (sc-13520; Santa Cruz Biotechnology, Inc., Santa Cruz, CA, USA) and anti-TIMP-1 at 1:250 (sc-21734; Santa Cruz Biotechnology, Inc., Santa Cruz, CA, USA) and mouse anti-TIMP-2 at 1:250 (sc-21735; Santa Cruz Biotechnology, Inc., Santa Cruz, CA, USA). All antibodies were thinned out with Diluent (Agilent Dako EnVision, Hovedstaden, Denmark). Slides were deparaffinized in three changes in xylene and rehydrated in a graded series of ethanol and distilled water. For antigen retrieval, the slides were immersed in a 0.01M citrate buffer at pH 6.0 and subjected to microwave heating for 10 min. Endogenous peroxidases were blocked by incubation in a Dual Endogenous Enzyme Block (Agilent Dako EnVision, Denmark) for 10 min. Afterwards, sections were incubated for 30 min at room temperature with the previously mentioned antibodies. Subsequently, sections underwent a 30 min incubation with a labeled polymer (Labeled Polymer HRP; Agilent Dako EnVision, Denmark), followed by a 10 min incubation with a substrate–chromogen complex containing 3,3′-diaminobenzidine (Agilent Dako EnVision, Denmark), resulting in the formation of a brown precipitate at the antigen site. Negative controls were prepared by excluding the primary antibody. After counterstaining with hematoxylin (Sigma-Aldrich, Poznań, Poland), the specimens were mounted using a permanent mounting medium. Prior to each incubation, tissues were washed twice in PBS for 5 min and subjected to a 5 min TBS bath. Every incubation was conducted in a humid chamber at room temperature. Staining was conducted in compliance with the manufacturer’s protocol. Upon the completion of the reaction, the slides were examined under a microscope and LAS software version 4.4 (Leica DM5000B, Wetzlar, Germany). To investigate the cardiac expressions of MMP-2, MMP-9, TIMP-1 and TIMP-2, samples were analyzed by the Cell Quant software version 2.3 (3DHISTECH Kft., Budapest, Hungary), which detected four levels of staining intensity and generated the area and perimeter expression, as well as object intensity of the IHC products (Figure 3). Six slides from each group were analyzed. Since our aim was to analyze tissue areas with the highest quality, small sections were selected, and scanned, the results were then grouped together and extracted. Two independent experts verified the results generated by the Cell Quant software.

### 2.7. Statistical Analysis

Analyses were calculated in the STATA 18 program. Continuous data were presented as medians with interquartile ranges (IQR). The Shapiro–Wilk test was applied to evaluate the normal distribution. Since the majority of the data were not normally distributed, the Kruskal–Wallis test and the post hoc Dunn’s test were performed. A *p*-value <0.05 was considered statistically significant.

## 3. Results

Two rats from the CRG cohort died before the end of the study; therefore, the analyses in the mentioned group were performed on tissues obtained from four animals only.

### 3.1. Morphology

**Figure 4:**
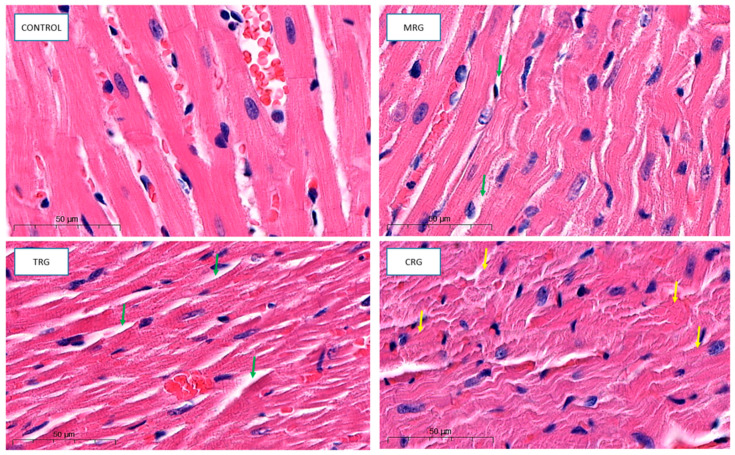
Representative pictures of cardiac tissue used for morphological analysis, HE staining, obj. Magnification ×50. Control: control group; MRG: mycophenolate mofetil + rapamycin + glucocorticosteroid group; TRG: tacrolimus rapamycin+glucocorticosteroid group; CRG: cyclosporine + rapamycin + glucocorticosteroid group (green arrows: dilated area with connective tissue; yellow arrows: altered cardiomyocytes).

Cardiomyocytes in the control group indicated proper architecture. Branches and intercalated disks were properly preserved. The nuclei of both cardiomyocytes and fibroblasts belonging to connective tissue were properly organized. The wall of blood vessels indicated a well structure, including endothelial cells. Cardiomyocytes from the MRG and TRG groups were altered. The area with connective tissue that separates each cardiac muscle was wider. Additionally, cells that formed CRG were also altered. The hypertrophy of myocytes, disturbed branches, and intercalated discs were observed (Figure 4).

### 3.2. Morphometry

**Figure 5:**
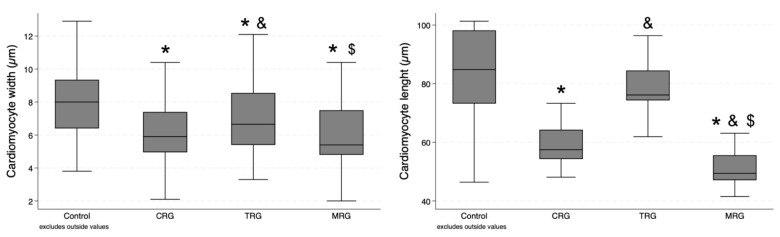
Comparative analysis of cardiomyocyte dimensions among immunosuppressive therapy groups. Chronic immunosuppression reduced cardiomyocyte width. CRG and MRG protocols reduced cardiomyocyte length. CRG—cyclosporine A + rapamycin + glucocorticosteroids; TRG—tacrolimus + rapamycin + glucocorticosteroids; MRG—mycophenolate mofetil + rapamycin + glucocorticosteroids. *—*p* < 0.05 vs. control; &—*p* < 0.05 vs. CRG; $—*p* < 0.05 vs. TRG (Dunn’s test). Middle line—median value; square—interquartile ranges; lower and upper lines—first and fourth quartile.

Sixty measurements of cardiomyocyte width and thirty measurements of cardiomyocyte length were performed in each group. Cardiomyocytes were the widest in the control group, and statistical significance was achieved when compared with each of the study groups (Table 1). Furthermore, cells in the TRG group were significantly longer than those from the CRG and MRG groups (*p* = 0.0241 and *p* = 0.0032, respectively). There was no significant difference between the CRG and MRG cohorts (Figure 5). In the case of cardiomyocyte length, the highest values were obtained in the control group, and statistical significance was achieved when it was compared to the CRG and MRG cohorts (Figure 5). Among the study groups, cellular length was the highest in the TRG group.

### 3.3. Vascular Density

**Figure 6:**
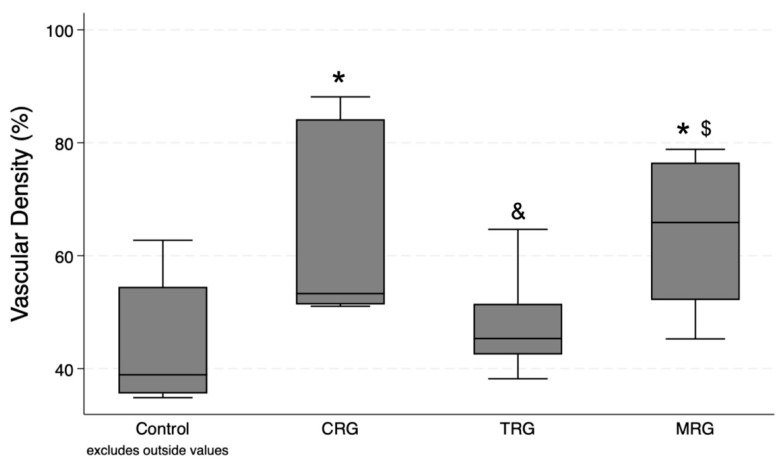
Evaluation of vascular density variation under different immunosuppressive regimens. CRG and MRG protocols increased vascular density. CRG—cyclosporine A + rapamycin + glucocorticosteroids; TRG—tacrolimus + rapamycin + glucocorticosteroids; MRG—mycophenolate mofetil + rapamycin + glucocorticosteroids. *—*p* < 0.05 vs. control; &—*p* < 0.05 vs. CRG; $—*p* < 0.05 vs. TRG (Dunn’s test). Middle line—median value; square—interquartile ranges; lower and upper lines—first and fourth quartile.

Ten measurements were performed in each group. According to the Kruskal–Wallis test, differences between the groups were statistically significant (*p* = 0.008). The post hoc test demonstrated significant differences between the CRG and MRG cohorts with the control group. Furthermore, the presence of blood vessels was significantly greater in these study groups than in the TRG cohorts, suggesting that immunosuppressant regiments composed of cyclosporine and mycophenolate mofetil, together with rapamycin and steroids, had the greatest impact on the formation of novel blood vessels (Figure 6).

### 3.4. Collagen Analysis

**Figure 7:**
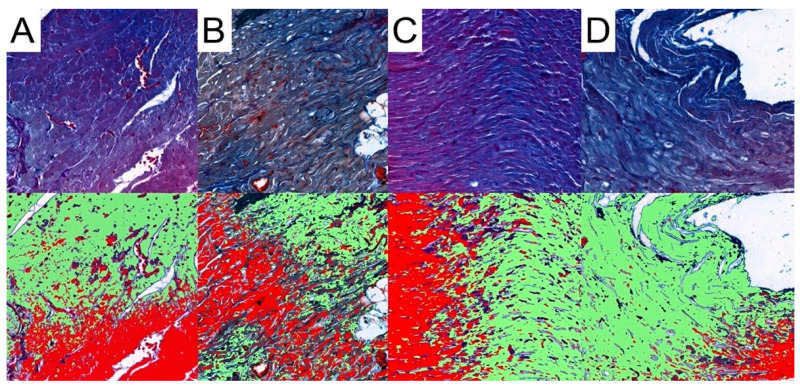
Representative pictures of presence of collagen fibers analyzed with the use of Pattern Quant. Green areas indicate the presence of collagen fibers and red areas indicate the absence of collagen fibers; Mallory trichrome staining of specimens from, obj. magnification ×20 (**A**): control group; (**B**): CRG group; (**C**): TRG group; and (**D**): MRG group.

**Figure 8:**
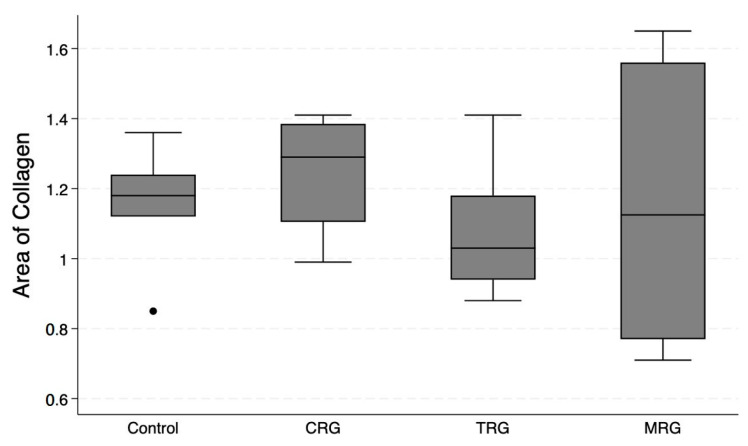
An assessment of the cardiac collagen deposition across immunosuppressive treatment groups. No statistically significant differences between groups were observed. CRG—cyclosporine A + rapamycin + glucocorticosteroids; TRG—tacrolimus + rapamycin + glucocorticosteroids; MRG—mycophenolate mofetil + rapamycin + glucocorticosteroids. Middle line—median value; square—interquartile ranges; lower and upper lines—first and fourth quartile.

Pattern Quant was used to analyze the whole sample sections, as demonstrated in Figure 2. Subsequently, we filtered the results to obtain only sections with the presence of collagen. Ultimately, the program analyzed 281,132, 212,086, 210,122, and 12,906 collagen-positive sections (Figure 7). Regarding the total area of collagen, the studied groups did not differ significantly (Kruskal–Wallis *p* = 0.675). Furthermore, Dunn’s post hoc test demonstrated no significant differences between the control and the studied groups, as well as between the CRG, TRG and MRG cohorts (Table 1, Figure 8). The highest values were noted in the CRG group (median 1.29 IQR 1.105–1.385). Overall, these findings suggest that the chronic administration of immunosuppressants did not alter cardiac collagen deposition.

### 3.5. Digital Analysis of Immunolocalization of the MMP-2/TIMP-2

**Figure 9:**
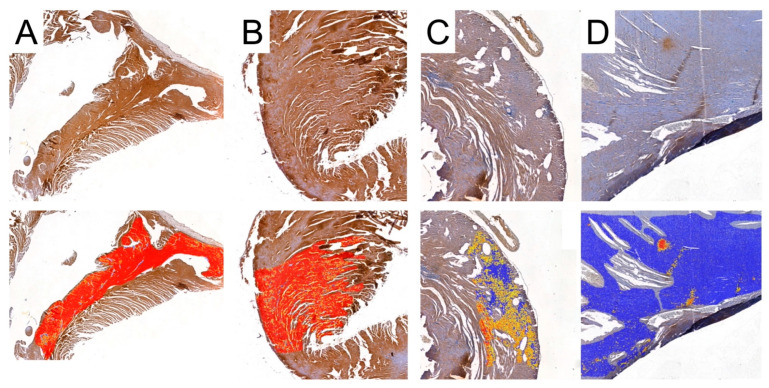
Representative pictures of MMP-2 immunoexpression analyses used by Cell Quant software. Red areas indicate the strongest immunoexpression; orange areas indicate intermediate immunoexpression; yellow areas indicate weak immunoexpression; blue areas indicate the absence of immunoexpression. (**A**): Control group; (**B**): CRG group (cyclosporine A + rapamycin + glucocorticosteroids); (**C**): TRG group (tacrolimus + rapamycin + glucocorticosteroids); and (**D**): MRG group (mycophenolate mofetil + rapamycin + glucocorticosteroids).

**Figure 10:**
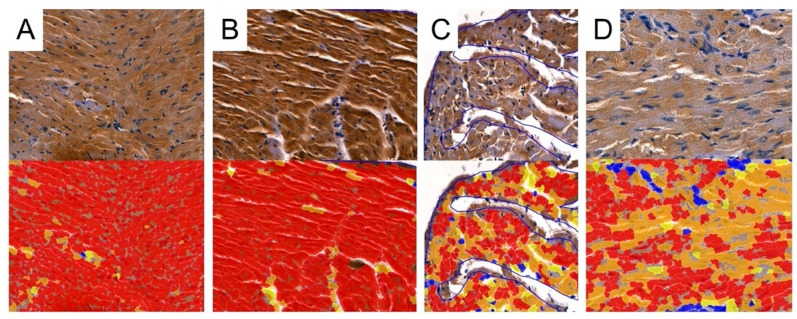
Representative pictures of TIMP-2 immunoexpression analyses used by Cell Quant software. Red areas indicate the strongest immunoexpression; orange areas indicate intermediate immunoexpression; yellow areas indicate weak immunoexpression; blue areas indicate the absence of immunoexpression. (**A**): Control group; (**B**): CRG group (cyclosporine A + rapamycin + glucocorticosteroids); (**C**): TRG group (tacrolimus + rapamycin + glucocorticosteroids); and (**D**): MRG group (mycophenolate mofetil + rapamycin + glucocorticosteroids).

**Figure 11:**
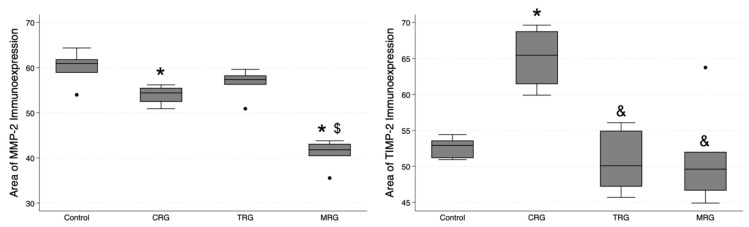
Comparative immunoexpression of MMP-2 and TIMP-2 amongst immunosuppressive regimens. CRG and MRG protocols reduced the immunostaining of MMP-2, while the CRG regiment enhanced the presence of TIMP-2. CRG—cyclosporine A + rapamycin + glucocorticosteroids; TRG—tacrolimus + rapamycin + glucocorticosteroids; MRG—mycophenolate mofetil + rapamycin + glucocorticosteroids, *—*p* < 0.05 vs. control; &—*p* < 0.05 vs. CRG; $—*p* < 0.05 vs. TRG (Dunn’s test). Middle line—median value; square—interquartile ranges; lower and upper lines—first and fourth quartile.

Cell Quant was used to analyze the immunostaining of cardiac samples (Figure 9 and Figure 10). The program classified selected areas into four categories, depending on the immunostaining intensity (0, +1, +2, and +3). To analyze differences in the total area with positive immunostaining, we selected regions marked as 1+, 2+ and 3+ for further analyses. The median area expressions of MMP-2 in the control, CRG, TRG, and MRG groups were 60.9; 54.4; 57.33; and 41.8, respectively. The total area of MMP-2 was significantly greater in the control group compared to the CRG and MRG groups (Table 1, Figure 11). Furthermore, greater MMP-2 immunostaining was observed in the TRG group than in the MRG group (*p* = 0.0027). Moreover, immunosuppressants dysregulated the expression profile of TIMP-2, which is an inhibitor of MMP-2. In the CRG group, the area of expression of TIMP-2 was significantly higher than the control (65.44 vs. 52.91, *p* = 0.0282). In addition, differences between CRG and the TRG and MRG groups were statistically significant as well. However, according to Dun’s test, the difference between the TRG and MRG cohorts was not significant (*p* = 0.3778).

### 3.6. Digital Analysis of Immunolocalization of the MMP-9/TIMP-1

**Figure 12:**
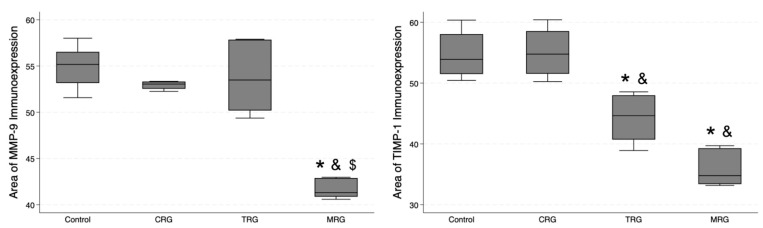
Differential expression of MMP-9 and TIMP-1 in response to immunosuppressive drugs. MRG protocol reduced the immunostaining of MMP-9, while chronic administration of TRG and MRG regiments decreased the presence of TIMP-1; CRG—cyclosporine A + rapamycin + glucocorticosteroids; TRG—tacrolimus + rapamycin + glucocorticosteroids; MRG—mycophenolate mofetil + rapamycin + glucocorticosteroids. *—*p* < 0.05 vs. control; &—*p* < 0.05 vs. CRG; $—*p* < 0.05 vs. TRG (Dunn’s test). Middle line—median value; square—interquartile ranges; lower and upper lines—first and fourth quartile.

In addition, we studied the expression of MMP-9 and TIMP-1 (Figure 12). The lowest area of MMP-9 immunoexpression was observed in the MRG group (median 41.31 IQR 40.87—42.89), and, importantly, it was significantly lower than the values obtained in the control and the other study groups (Table 1). Regarding TIMP-1, an MMP-9 inhibitor, the control group and CRG did not differ significantly (*p* = 0.5), while greater immunostaining was observed in the control group compared to the TRG (*p* < 0.0131) and the MRG (*p* < 0.0001) groups. Among the study groups, the immunostaining of TIMP-1 was the greatest in rats receiving the CRG protocol (*p* = 0.0234 vs. TRG; *p* = 0.0006 vs. MRG, Table 1). Taken together, these results indicate that the expression of MMP-9 was reduced in rats treated with MRG, while that of TIMP-1 was reduced in animals treated with TRG and MRG protocols.

## 4. Discussion

Immunosuppressive agents have various mechanisms of action. Cyclosporine A and tacrolimus inhibit the activity of calcineurin: a Ca^2+^-dependent serine/threonine phosphatase associated with the signaling pathway of interleukin-2 (IL-2). Specifically, the suppression of calcineurin prevents the activity of the nuclear factor of the activated T cell (NFAT1c) transcription factor, which ultimately inhibits the production of IL-2, which is a cytokine that plays an important role in T cell activation and the stimulation of immune response [15]. Mycophenolate is a prodrug of mycophenolic acid, an inhibitor of the inosine-5′-monophosphate dehydrogenase, and an enzyme taking part in the synthesis of nucleotides [16]. Rapamycin, also known as sirolimus, targets and inhibits mTOR, which suppresses cell cycle progression [17]. Various treatment strategies incorporating immunosuppressive agents are used in clinical practice in transplant recipients. These drugs are known to induce serious adverse effects, and investigating the mechanisms and pathways affected by these drugs is of great interest to improve future treatment strategies.

In this study, we analyzed the impact of several treatment regimens on the cardiomyocyte morphology and morphometry, as well as on the presence of collagen and the immunolocalization of MMPs and TIMPs in the cardiac tissue of rats. Our results indicated that all examined combinations of immunosuppressive drugs affected the morphology of cardiomyocytes. Nonetheless, it seems that the CRG protocol caused the most significant alterations. In line with our finding, in the analysis by Surówka et al., the authors investigated the impact of immunosuppressants on the morphology of aortas. In their study, they observed that the most profound alterations occurred in the CRG and TMG (tacrolimus, mycophenolate mofetil, steroids) cohorts. Precisely, treatment with CRG changed the shape of vascular smooth muscle cells and induced the folding of the endothelium [14]. Additionally, our analysis regarding the morphometry indicated that the widest cardiomyocytes were found in the control group, while the thinnest cells were observed in the MRG cohort. Perhaps a combination of mycophenolate mofetil with rapamycin affects the proteins included in the junctions, such as desmosomes (desmogleins and desmoplakins) or adherent junctions (cadherins). Of note, mycophenolate mofetil reduces the expression of genes associated with smooth muscle cell proliferation and attenuates intimal hyperplasia [18]. Perhaps mycophenolate mofetil could also impact the proliferation of cardiomyocytes by altering the expression of adhesion molecules.

We found that immunosuppressive therapy composed of cyclosporine/tacrolimus/mycophenolate, rapamycin, and steroids did not alter collagen deposition and degeneration in the hearts. Conflicting results were previously published regarding the impact of cyclosporine on cardiac architecture. In an early in vitro study, the drug did not alter the production of collagen in cardiac fibroblasts [19]. On the contrary, in an in vivo experiment incorporating rats, subcutaneous injections of cyclosporine for 21 days significantly increased the cardiac collagen volume [20].

We further evaluated whether immunosuppressants altered the immunoexpression of enzymes involved in extracellular matrix remodeling. Specifically, we analyzed the immunolocalization of MMPs and TIMPs. The studied protocols changed the expression profile of MMPs/TIMPs differently. For instance, immunosuppressants (CRG and MRG) significantly reduced the immunoexpression of MMP-2. Simultaneously, a higher presence of TIMP-2 was noted in the CRG cohort compared to the control animals. Moreover, the immunostaining was greater in the CRG group compared with the TRG and MRG cohorts. Perhaps the chronic application of drugs included in the CRG strategy might enhance collagen deposition by downregulating MMP-2 and upregulating TIMP-2; however, other regulatory mechanisms and pathways mediate collagen remodeling. The results induced by the other strategies might involve different processes or depend on the cellular context. Regarding MMP-9, only the MRG protocol was associated with weaker immunostaining, while in the case of TIMP-1, significant differences were observed only in the TRG and MRG cohorts.

Several studies demonstrated that immunosuppressants dysregulate the MMP/TIMP balance in organs. Nevertheless, the impact may depend on the dose, cell type, as well as isoforms of the enzymes. To begin with, Berthier et al. studied the effect of cyclosporine in rats that underwent heart transplantation. The authors compared the three groups of animals without transplantation, after transplantation and untreated, and a cohort of animals after the procedure received the calcineurin inhibitor. The authors observed that *MMP2* gene expression in the hearts of rats that experienced acute rejection was significantly higher than in rats without transplantation. Treatment with cyclosporine was associated with a significant reduction in *MMP2* expression. However, it was still higher than in the control group [21]. In another study, rats were subcutaneously injected with cyclosporine-A for 21 days. The treatment expanded the presence of collagen fibers and promoted the expression of MMP-2 and vascular endothelial growth factor [22]. Furthermore, the stimulation of endothelial cells with cyclosporine increased the activity of several MMPs (-1, -3, -8, -9, -13), and it decreased the activity of MMP-2 [23]. Several studies examined the impact of the drug on gingival fibroblasts since gingival overgrowth is one of the potential adverse effects that may occur in patients treated with this calcineurin inhibitor. These studies also demonstrated the significant impact of this drug on the expression of cartilage-degrading enzymes [24,25]. Tacrolimus, a more potent calcineurin inhibitor, has been found to suppress collagen production induced by transforming growth factor-beta 1 (TGF-β1) [26]. Regarding other agents, Alnsasra and collaborators studied the impact of immunosuppressive therapy on myocardium in 100 heart transplant recipients. The authors divided these patients into a group treated with calcineurin inhibitors and the second group receiving sirolimus and analyzed the progression of fibrosis up to three years after the procedure. Contrary to our results, the authors found a significant stimulation of fibrosis in the first cohort (*p* = 0.04) and a trend towards increased fibrosis in the second group (*p* = 0.07) [27]. In animal models of cardiac ischemia-reperfusion injury and dilated cardiomyopathy, rapamycin suppressed the formation of fibrosis [28,29]. The combination of cyclosporine with sirolimus synergistically induced the production of TGF-*β* and suppressed the gene expression of *MMP-9* in human mesangial cells [30].

Some of the discussed studies may demonstrate conflicting results. However, these differences may result from technical issues. Berthier and colleagues [21] examined hearts after 5 days of transplantation, while Bianchi et al. [22] analyzed tissues after cyclosporine treatment for 21 days. Meanwhile, animals in the present study did not undergo a transplantation procedure, and they might have been receiving immunosuppressants for 6 months. Additionally, in the present study, a combination of drugs was used, which more reliably simulated the treatment strategies of graft recipients. In a similar study by Surówka et al., the impact of the same regiments on rat aortas was investigated. Cohorts treated with TRG and CRG demonstrated a significantly greater presence of collagen compared to the control. Regarding the expression of MMPs/TIMPs, CRG was associated with increased, while MRG was associated with decreased expression of MMP-2. There were no significant differences between the controls and CRG, TRG, and MRG in the case of MMP-9 expression. CRG enhanced, while MRG suppressed the expression of TIMP-2 and increased the immunostaining of TIMP-1, as observed in the TRG and MRG cohorts [14].

In this study, we incorporated a unique AI-based software to examine the presence of collagen and the expression of IHC products. It provides a rapid and comprehensive analysis of large or whole tissue sections, thus increasing reliability and accuracy. In recent years, the implementation of AI has provided significant improvements in reading histological and histopathological samples. In clinical settings, AI tools have been examined in studies analyzing fibrosis [31], as well as in oncological studies on cancer diagnosis [32].

This study cannot be considered without certain limitations. As previously mentioned in the methods section, we scanned and analyzed available tissues of the highest quality. Consequently, studied groups differed in the case of the number of observations, written here as counts. Due to technical issues and variations in the distribution of staining, it was difficult to incorporate standardized scanning areas.

## 5. Conclusions

To conclude, the findings in this study suggest that immunosuppressive regimens did not alter collagen deposition. However, combinations of immunosuppressants dysregulated the MMP/TIMP homeostasis. Further studies are required to investigate the mechanisms of immunosuppressants on the fibrotic mechanisms occurring in hearts. The current study undoubtedly constitutes a unique one since, to the best of our knowledge, no other study analyzed the impact of three-drug immunosuppressive protocols on morphology, morphometry, vascular density, and MMP/TIMP balance using the AI methods. AI-based tools provide a reliable, accurate, rapid, and objective analysis of tissue samples, which represents an exciting approach in future histopathological studies. However, the results of the analyses generated by these tools need to be verified by experienced specialists.

## Figures and Tables

**Table 1:** Summary of the impact of different immunosuppressive protocols on cardiomyocyte morphometry, vascular density, the presence of collagen, as well as immunostaining with MMPs/TIMPs. Data are presented as medians and IQRs.

Variable	Control (*n* = 6)	CRG (*n* = 4)	TRG (*n* = 6)	MRG (*n* = 6)	*p*-Value (Kruskal–Wallis)	*p*-Value (vs. Control)	P1	P2	P3
Cardiomyocyte width	8 (6.4; 9.35)	5.9 (4.95; 7.4)	6.65 (5.4; 8.55)	5.4 (4.8; 7.5)	0.0001	CRG: <0.0001 TRG: 0.0129 MRG: 0.0113	0.0241	0.2271	0.0032
Cardiomyocyte length	84.8 (73.2; 98.2)	57.5 (54.3; 64.3)	76.15 (74.3; 84.5)	49.4 (47.1; 55.6)	0.0001	CRG: <0.0001 TRG: 0.2421 MRG: <0.0001	<0.0001	0.0177	<0.0001
Vascular density	38.9 (35.59; 54.5)	53.28 (51.34; 84.17)	45.33 (42.5; 51.47)	65.87 (52.15; 76.49)	0.008	CRG: 0.0062 TRG: 0.2579 MRG: 0.0019	0.032	0.3483	0.0125
Collagen area	1.18 (1.12; 1.24)	1.29 (1.105; 1.385)	1.03 (0.94; 1.18)	1.125 (0.77; 1.56)	0.675	CRG: 0.2555 TRG: 0.2592 MRG: 0.4645	0.1084	0.2305	0.2888
MMP-2 area	60.9 (58.85; 61.84)	54.4 (52.42; 55.52)	57.33 (56.21; 58.27)	41.8 (40.41; 43.12)	0.0009	CRG: 0.0239 TRG: 0.1284 MRG: 0.0000	0.1674	0.0641	0.0027
TIMP-2 area	52.91 (51.14; 53.59)	65.44 (61.43; 68.77)	50.08 (47.18; 54.94)	49.61 (46.63; 52.02)	0.0214	CRG: 0.0282 TRG: 0.2118 MRG: 0.1332	0.0043	0.0019	0.3778
MMP-9 area	55.18 (53.17; 56.53)	53.06 (52.54; 53.32)	53.49 (50.2; 57.85)	41.31 (40.87; 42.89)	0.004	CRG: 0.2132 TRG: 0.2385 MRG: 0.0003	0.4368	0.0117	0.0033
TIMP-1 area	53.9 (51.49; 58.04)	54.75 (51.53; 58.54)	44.64 (40.71; 47.99)	34.78 (33.4; 39.29)	0.0005	CRG: 0.5 TRG: 0.0131 MRG: 0.0001	0.0234	0.0006	0.0774

P1—CRG vs. TRG; P2—CRG vs. MRG; P3—TRG vs. MRG, Dunn’s test. CRG—cyclosporine, rapamycin, glucocorticosteroids; TRG—tacrolimus, rapamycin, glucocorticosteroids; MRG—mycophenolate, rapamycin, glucocorticosteroids.

## Data Availability

The data presented in this study are available upon reasonable request from the corresponding author.
